# ﻿Two new genera and five new species of Corinnidae Karsch, 1880 (Arachnida, Araneae) from China and Vietnam

**DOI:** 10.3897/zookeys.1165.102672

**Published:** 2023-05-30

**Authors:** Ying Lu, Chang Chu, Zixuan Lin, Dinh-Sac Pham, Shuqiang Li, Zhiyuan Yao

**Affiliations:** 1 College of Life Science, Shenyang Normal University, Shenyang 110034, Liaoning, China Shenyang Normal University Shenyang China; 2 Institute of Zoology, Chinese Academy of Sciences, Beijing 100101, China Institute of Zoology, Chinese Academy of Sciences Beijing China; 3 Vietnam National Museum of Nature (VNMN), Vietnam Academy of Science and Technology (VAST), 18 Hoang Quoc Viet, Cau Giay, Hanoi, Vietnam Vietnam National Museum of Nature Hanoi Vietnam

**Keywords:** biodiversity, morphology, new taxa, taxonomy, tropics

## Abstract

Six species of the family Corinnidae Karsch, 1880 are described from China and Vietnam. *Fengzhen***gen. nov.** is erected to accommodate *F.mengla***sp. nov.** (♂♀) from China; *Peng***gen. nov.** is erected to accommodate *P.birmanicus* (Thorell, 1897), **comb. nov.**, *P.borneensis* (Yamasaki, 2017), **comb. nov.** and *P.taprobanicus* (Simon, 1897), **comb. nov.**, transferred from *Sphecotypus* O. Pickard-Cambridge, 1895. Further new species described include *Allomedmassatamdao***sp. nov.** (♂), *Echinaxbaisha***sp. nov.** (♂), *Medmassalingshui***sp. nov.** (♂), and *Spinirtashaoguan***sp. nov.** (♂). The male of *P.birmanicus* is described for the first time.

## ﻿Introduction

The spider family Corinnidae comprises of 843 species in 72 genera, distributed in Asia, Africa, Australia, and the Americas (WSC 2023). Only one species, *Castianeirabadia* (Simon, 1877), is distributed in Europe (WSC 2023). They are usually caught in pitfall traps in open forest, heathland, and desert (Raven 2015). The corinnid subfamily Castianeirinae Reiskind, 1969 comprises a large number of ant-mimicking spiders, with a few mimicking wasps or other spiders (Cushing 2012). Reiskind (1969) merged 15 genera from the subfamily Micariinae Platnick & Shadab, 1988 of the family Clubionidae Wagner, 1887 into Castianeirinae based on the structure of the fovea and genitals, as well as the degree of “ant-mimicking”. He referred to *Castianeira* Keyserling, 1879 as the type genus of Castianeirinae and transferred it to Corinnidae. Currently, 16 castianeirine genera are recognized from the forests of Southeast Asia. These are: *Aetius* O. Pickard-Cambridge, 1897, *Allomedmassa* Dankittipakul & Singtripop, 2014, *Apochinomma* Pavesi, 1881, *Castianeira* Keyserling, 1879, *Castoponera* Deeleman-Reinhold, 2001, *Coenoptychus* Simon, 1885, *Copa* Simon, 1886, *Corinnomma* Karsch, 1880, *Echinax* Deeleman-Reinhold, 2001, *Fluctus* Jin & Zhang, 2020, *Humua* Ono, 1987, *Medmassa* Simon, 1877, *Paramedmassa* Jin, Zhang & Zhang, 2019, *Pranburia* Deeleman-Reinhold, 1993, *Serendib* Deeleman-Reinhold, 2001 and *Sphecotypus* O. Pickard-Cambridge, 1895 (WSC 2023).

According to Jin et al. (2019), most genera of Castianeirinae are ant mimics and all members share the presence of the following diagnostic characters: 1) male palpal bulb pyriform, provided with an apical embolus, and lacking a conductor and median apophysis, and 2) female genitalia without separate bursa, with the spermatheca and bursa united into one elongate, sclerotized, and partly folded structure (Deeleman-Reinhold 2001; Raven 2015). However, some genera of Castianeirinae are neither mimics nor exhibit the above genitalic characteristics, such as *Allomedmassa* and *Medmassa*. The latest molecular evidence shows that *Allomedmassa* may not belong to Castianeirinae (Wheeler et al. 2017). Ramírez (2014) conducted a phylogenetic analysis and found that the genus *Allomedmassa* (sub cf. *Medmassa* THA) appears as the sister group of Corinninae and Castianeirinae, respectively. Except for the genera mentioned above, *Spinirta* Jin & Zhang, 2020, is not a castianeirine, corinnine, nor a “*Pronophaea* group” species based on the morphological evidence: ear-shaped or spoon-shaped RTA with thick spines; bifurcated embolus with file-like grooves; copulatory openings separated or fused into a large atrium (Jin and Zhang 2020).

The goals of the present paper are the description of two new genera: *Fengzhen* gen. nov. and *Peng* gen. nov.; the description of five new species: *Allomedmassatamdao* sp. nov., *Echinaxbaisha* sp. nov., *Fengzhenmengla* sp. nov., *Medmassalingshui* sp. nov., and *Spinirtashaoguan* sp. nov.; and the first description of male *Pengbirmanicus* (Thorell, 1897) comb. nov.

## ﻿Materials and methods

Specimens were examined and measured with a Leica M 205C stereomicroscope. Left male pedipalps were photographed and drawn. Epigynes were photographed before dissection. Vulvae were treated in a 10% warm solution of potassium hydroxide (KOH) to dissolve soft tissues before illustration. Images were captured with a Canon EOS 750D wide zoom digital camera (24.2 megapixels) mounted on the stereomicroscope mentioned above and assembled using Helicon Focus v. 3.10.3 image stacking software (Khmelik et al. 2005). All measurements are given in millimetres (mm). Leg measurements are shown as: total length (femur, patella, tibia, metatarsus, tarsus), missing data were coded as ‘–’. Leg segments were measured on their dorsal side. The species distribution map was generated with ArcGIS v. 10.2 (ESRI, Inc.). Spination is variable, even within the same species or individual, the ventral spines always appear in pairs and are sometimes important diagnosis, therefore, arrangement of spines of other views are not included in species descriptions. The specimens studied are preserved in 75% ethanol and deposited in the Institute of Zoology, Chinese Academy of Sciences (IZCAS) in Beijing, China.

Terminology and taxonomic descriptions refer to Jin et al. (2019) and Zhang et al. (2023).

The following abbreviations are used in the descriptions:

**AER** anterior eye row;

**ALE** anterior lateral eye;

**AME** anterior median eye;

**CRW** width of cephalic region at PLE;

**MOA** median ocular area;

**PER** posterior eye row;

**PLE** posterior lateral eye;

**PME** posterior median eye.

## ﻿Taxonomy

### ﻿Family Corinnidae Karsch, 1880

#### ﻿Genus Allomedmassa

Taxon classificationAnimaliaAraneaeCorinnidae

Dankittipakul & Singtripop, 2014

5AE099AD-33BA-5AE1-BDCE-08B618D0AEB7

##### Type species.

*Allomedmassamae* Dankittipakul & Singtripop, 2014 from Thailand.

##### Composition.

The genus is endemic to Southeast Asia, and currently contains five species: *A.bifurca* Jin, Zhang & Zhang, 2019 (♂) from China, *A.crassa* Jin, Zhang & Zhang, 2019 (♂) from China, *A.deelemanae* Dankittipakul & Singtripop, 2014 (♂) from Malaysia, *A.mae* (♂♀) from Thailand and China, and *A.matertera* Jin, Zhang & Zhang, 2019 (♀) from China.

#### ﻿ Allomedmassa tamdao

Taxon classificationAnimaliaAraneaeCorinnidae

Lu & Li sp. nov.

D0DA4820-1270-5F91-A6A3-28560D96FE28

https://zoobank.org/B2D688F7-0B2D-4EAA-942E-F457CFE8F483

Figs 1 , 2

##### Type material.

***Holotype***: 1♂ (IZCAS-Ar 44416), **Vietnam**, Vinh Phuc, Tam Dao National Park, 21°29.428′N, 105°37.008′E, 1077 m, Sieving in leaf litter, 21 August 2007, D.S. Pham leg.

##### Etymology.

The specific name refers to the type locality and is a noun in apposition.

##### Diagnosis.

**Figure 1.**
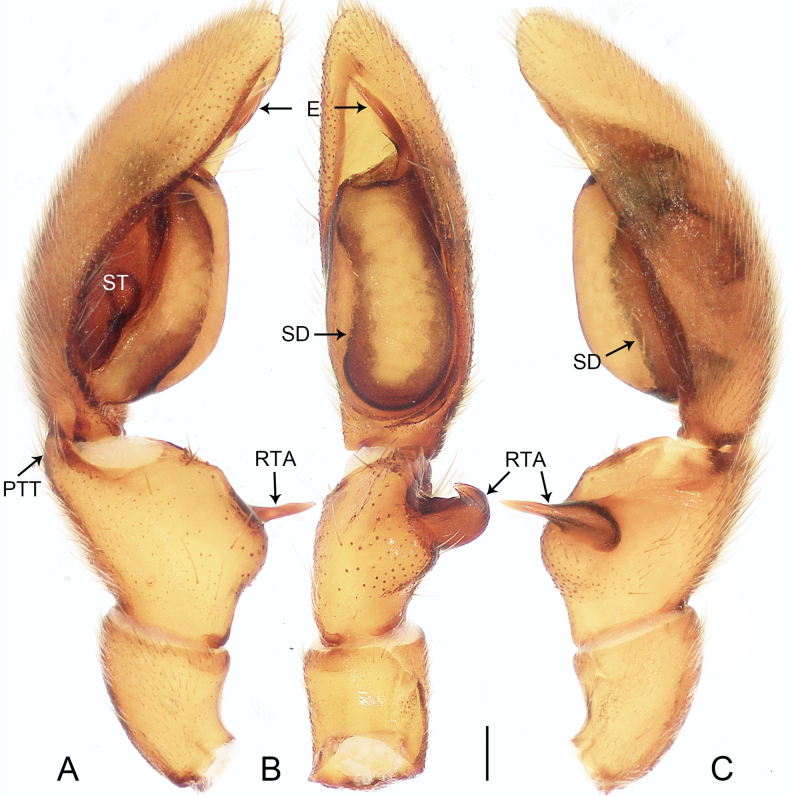
*Allomedmassatamdao* sp. nov., holotype male **A–C** palp **A** prolateral view **B** ventral view **C** retrolateral view. Abbreviations: E = embolus, PTT = prolateral tibial tubercle, RTA = retrolateral tibial apophysis, SD = sperm duct, ST = subtegulum. Scale bar: 0.20 mm.

**Figure 2.**
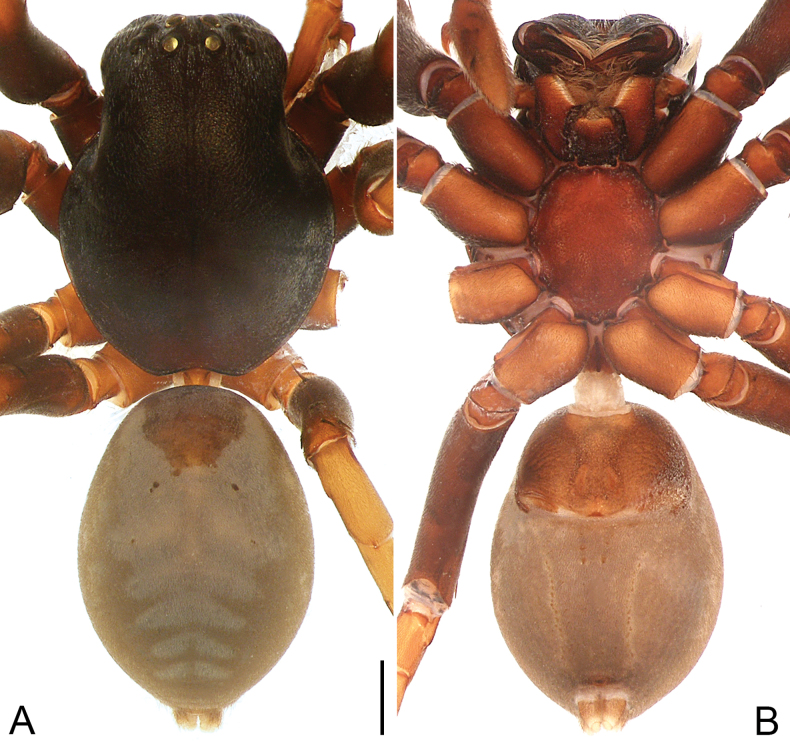
*Allomedmassatamdao* sp. nov., holotype male **A, B** habitus **A** dorsal view **B** ventral view. Scale bar: 1.00 mm.

The new species resembles *A.bifurca* Jin, Zhang & Zhang, 2019 (cf. Figs 1, 2 and Jin et al. 2019: 462, figs 3A–G, 4A–C) in that the males have a similar triangular prolateral tibial tubercle (Fig. 1A). Males can be distinguished by the slightly curved and spine-shaped embolus, which is more slender (Fig. 1B; vs. S-shaped embolus, strong, with fine dorsal branch near tip, originating from the base of the embolus and shorter than the embolus), by the tegulum being slightly convex on the retrolateral side (Fig. 1C; vs. tegulum strongly convex on the retrolateral side), and by the retrolateral tibial apophysis strong, with a wide base and a sharp, strongly curved tip (Fig. 1A–C; vs. retrolateral tibial apophysis relatively slender, spine-shaped and slightly curved). Female unknown.

**Figure 3.**
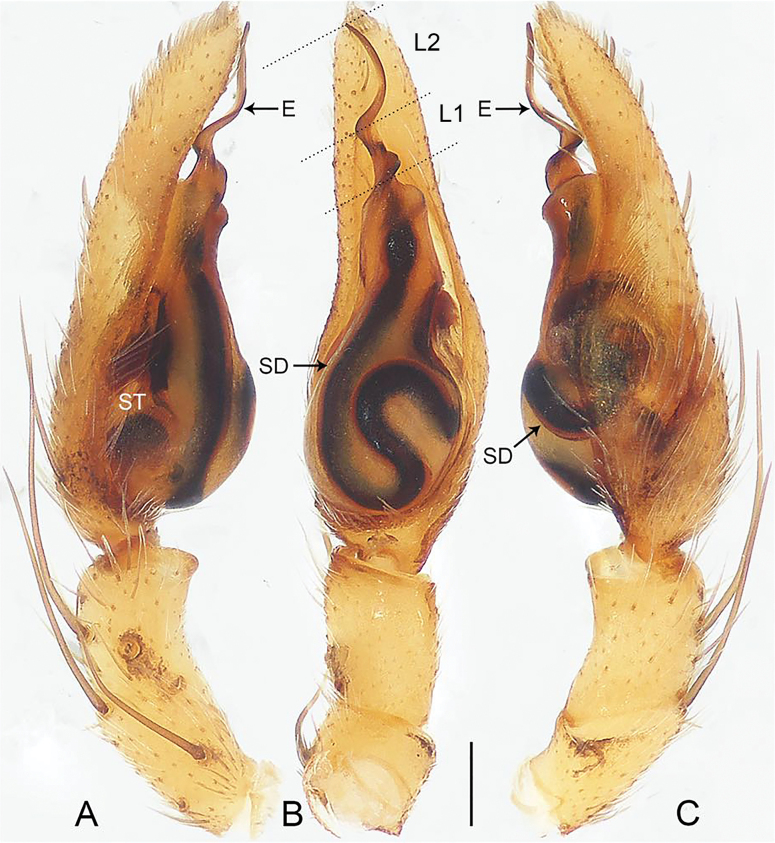
*Echinaxbaisha* sp. nov., holotype male **A–C** palp **A** prolateral view **B** ventral view **C** retrolateral view. Abbreviations: E = embolus, L1 = length 1, L2 = length 2, SD = sperm duct, ST = subtegulum. Scale bar: 0.20 mm.

**Figure 4.**
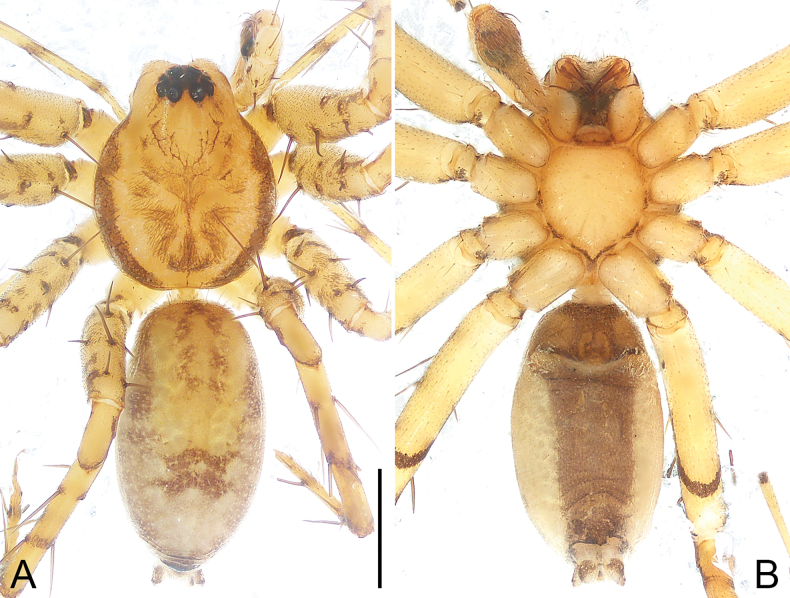
*Echinaxbaisha* sp. nov., holotype male **A, B** habitus **A** dorsal view **B** ventral view. Scale bar: 1.00 mm.

##### Description.

**Male** (**holotype**; Fig. 2A, B). Total body length 9.72: carapace 4.83 long, 3.75 wide; abdomen 4.89 long, 3.23 wide. Carapace black, obviously convex, with rough surface, highest before fovea; thoracic region almost round and cephalic region long and parallel-sided; widest at coxae II, gradually narrowing backwards, strongly concave at posterior margin before pedicel; radial and cervical grooves indistinct; fovea longitudinal, short. Diameters of eyes: AME 0.26, ALE 0.18, PME 0.22, PLE 0.22. Eye interdistances: AME–AME 0.18, AME–ALE 0.17, PME–PME 0.36, PME–PLE 0.36, AME–PME 0.26, ALE–PLE 0.16. CRW/carapace width = 0.70. MOA 0.71 long, front width 0.67, back width 0.74. Clypeus height narrower than diameter of AME. Chilum present, single, triangular, sclerotized, and brown. Chelicerae same color as carapace, concave at distal end dorsally; with three promarginal teeth, five retromarginal teeth. Endites and labium dark brown, longer than wide; endites subapically with membranous area, apical margin with long, curved setae. Labium 0.86 long, 0.68 wide. Sternum brown, shield-shaped, precoxal triangles and intercoxal sclerites present. Sternum 2.24 long, 1.83 wide. Legs dark brown to brown. Measurements of legs: I 15.25 (4.26, 1.82, 3.74, 3.44, 1.99), II 14.41 (4.12, 1.71, 3.40, 3.31, 1.87), III 11.83 (3.24, 1.53, 2.62, 2.97, 1.47), IV 15.23 (4.09, 1.59, 3.65, 4.25, 1.65). Leg spination: tibiae I–II with four pairs of ventral spines, III–IV with two pairs of ventral spines; metatarsi I and II with two pairs of ventral spines, III and IV with three pairs of ventral spines. Abdomen ovoid, dark grey, with brown dorsal scutum anteriorly, posteriorly with several light grey chevrons; venter anteriorly with brown, rectangular scutum, posteriorly dark grey. Spinnerets grey.

***Palp*** (Fig. 1A–C). Tibia with ventral surface flat and slanting, not forming a hump, with triangular prolateral tibial tubercle; retrolateral tibial apophysis well developed, with wide base and sharp end, only curved at end. Cymbium tip conical. Tegulum slightly flattened basally, slightly convex on retrolateral side, 3/5 length of cymbium, with U-shaped sperm duct. Subtegulum exposed prolaterally. Embolus slender, spine-shaped, and slightly curved.

##### Distribution.

Vietnam (Vinh Phuc, type locality; Fig. 14).

#### ﻿Genus Echinax

Taxon classificationAnimaliaAraneaeCorinnidae

Deeleman-Reinhold, 2001

8DCF7367-1B02-500B-B378-3A1E1FF025CB

##### Type species.

*Copaoxyopoides* Deeleman-Reinhold, 1995 from Indonesia.

##### Composition.

The genus includes 12 species mainly distributed in Africa and Asia. Only five species are distributed in Southeast Asia: *E.anlongensis* Yang, Song & Zhu, 2004 (♀) from China, *E.bosmansi* (Deeleman-Reinhold, 1995) (♀) from Indonesia, *E.javana* (Deeleman-Reinhold, 1995) (♂) from Indonesia, *E.oxyopoides* (Deeleman-Reinhold, 1995) (♂♀) from China, Indonesia and Borneo, and *E.panache* Deeleman-Reinhold, 2001 (♂♀) from China, India, and Thailand.

#### ﻿ Echinax baisha

Taxon classificationAnimaliaAraneaeCorinnidae

Lu & Li sp. nov.

3EE49DED-B48B-5CDD-B7BC-B4AC25478797

https://zoobank.org/13E3822C-A895-42EF-8CE7-97C0803DFB48

Figs 3 , 4

##### Type material.

***Holotype***: 1♂ (IZCAS-Ar 44417), **China**, Hainan, Baisha, Yinggeling Nature Reserve, Yinggezui Protection Station, 19°03.049′N, 109°33.751′E, 663 m, hand catch in leaf litter, 25 August 2010, G. Zheng leg. ***Paratypes***: 2♂ (IZCAS-Ar 44418, 44419), **China**, Hainan, Ledong, Jianfengling National Forest Park, Mingfenggu, 18°44.658′N, 108°50.435′E, 1017 m, 18 August 2010, G. Zheng leg.

##### Etymology.

The specific name refers to the type locality and is a noun in apposition.

##### Diagnosis.

The new species resembles *E.hesperis* Haddad, 2012 (cf. Figs 3, 4 and Haddad 2012: 43, figs 3–4, 46, 56–59), as the males have a similar sperm duct (Fig. 3A–C). Males can be distinguished by the embolus that is relatively more curved apically (Fig. 3A–C; vs. embolus relatively slightly curved apically), and by the base of the embolus almost straight (Fig. 3B; vs. base of the embolus curved). Female unknown.

##### Description.

**Male** (**holotype**; Fig. 4A, B). Total body length 4.31: carapace 1.88 long, 1.54 wide; abdomen 2.43 long, 1.29 wide. Carapace yellowish, with brown marginal bands, and brown median patterns. Fovea brown, longitudinal and shallow. Diameters of eyes: AME 0.11, ALE 0.05, PME 0.08, PLE 0.08. Eye interdistances: AME–AME 0.09, AME–ALE 0.03, PME–PME 0.15, PME–PLE 0.08, AME–PME 0.18, ALE–PLE 0.06. CRW/carapace width = 0.53. MOA 0.33 long, front width 0.28, back width 0.29. Chelicerae same color as carapace; with two promarginal teeth, two retromarginal teeth. Endites yellowish, longer than wide, apical margin with long, curved setae. Labium yellowish, 0.20 long, 0.36 wide. Sternum yellowish, shield-shaped, with marginal dark stripes posteriorly. Sternum 0.96 long, 0.89 wide. Legs yellowish, with few dorsal spots and rings. Measurements of legs: I 6.38 (1.92, 0.63, 1.60, 1.52, 0.71), II 6.10 (1.89, 0.59, 1.46, 1.49, 0.67), III 6.05 (1.74, 0.62, 1.40, 1.59, 0.70), IV 7.26 (2.06, 0.64, 1.68, 2.10, 0.78). Leg spination: tibiae I–II, IV with 3 pairs of ventral spines, III with two pairs of ventral spines; metatarsi I–III with two pairs of ventral spines, IV with three pairs of ventral spines. Abdomen cylindrical, yellowish, with two long brown median stripes in anterior part, marginal light brown bands and brown marks in posterior part; venter yellowish, anteriorly with brown scutum, with wide, longitudinal brown stripe. Spinnerets yellowish with brown patterns.

***Palp*** (Fig. 3A–C). Tibia without retrolateral apophysis. Cymbium elongate, with three pairs of stout spatulate setae on dorsal surface. Tegulum long, 3/5 length of cymbium, with distinct, sinuous sperm duct. Embolus slender and screwed, making two turns, short and straight basally, curved and tapered apically, length of embolus: L1/L2 = 1/2.

##### Variation.

Paratype males: total body length 3.72–4.87.

##### Distribution.

China (Hainan, type locality; Fig. 14).

#### ﻿Genus Fengzhen

Taxon classificationAnimaliaAraneaeCorinnidae

Lu & Li gen. nov.

5341A487-E87F-5930-AD00-86343BA19F2B

https://zoobank.org/2B5F0371-91D6-4286-A00E-B272FF42397E

##### Type species.

*Fengzhenmengla* Lu & Li, sp. nov.

##### Composition.

Monotypic.

##### Etymology.

The generic name is dedicated to the late Chinese arachnologist Fengzhen Wang (1906–1978). Gender is masculine.

##### Diagnosis.

**Figure 5.**
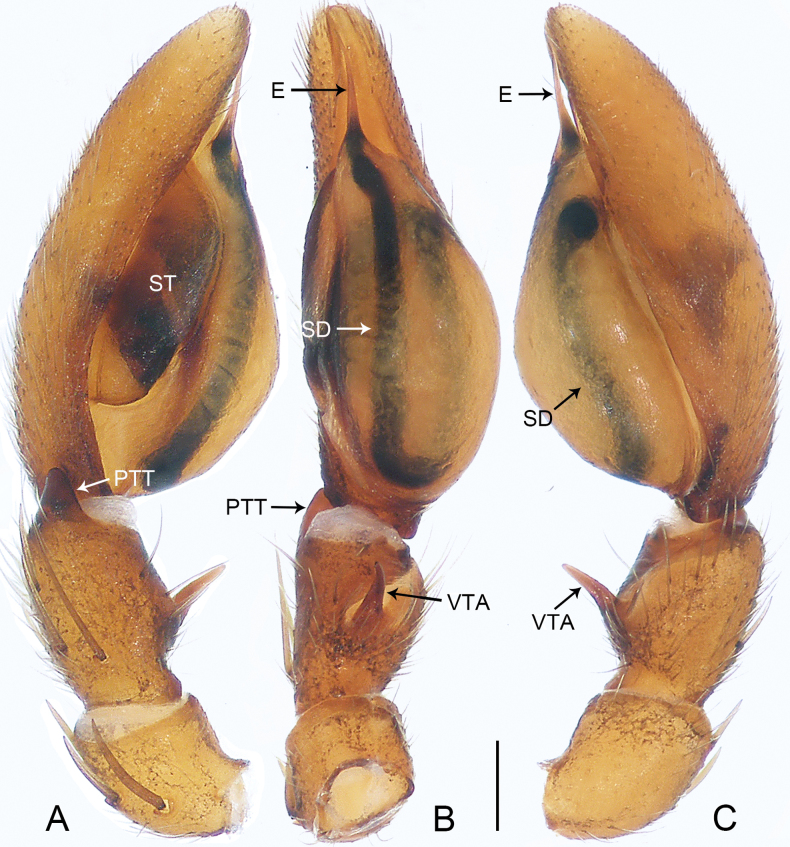
*Fengzhenmengla* sp. nov., holotype male **A–C** palp **A** prolateral view **B** ventral view **C** retrolateral view. Abbreviations: E = embolus, PTT = prolateral tibial tubercle, SD = sperm duct, ST = subtegulum, VTA = ventral tibial apophysis. Scale bar: 0.20 mm.

**Figure 6.**
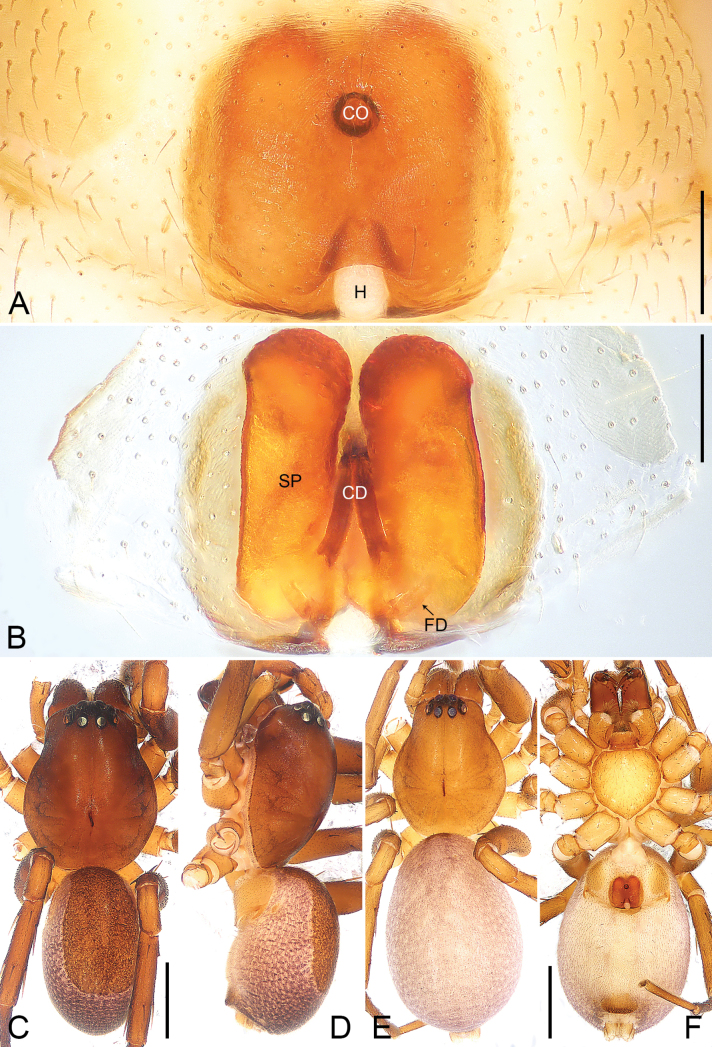
*Fengzhenmengla* sp. nov., holotype male (**C, D**) and paratype female (**A, B, E, F**) **A** epigyne, ventral view **B** vulva, dorsal view **C** habitus, dorsal view **D** habitus, lateral view **E** habitus, dorsal view **F** habitus, ventral view. Abbreviations: CD = copulatory duct, CO = copulatory opening, FD = fertilization duct, H = hood, SP = spermathecae. Scale bars: 0.20 mm (**A, B**); 1.00 mm (**C–F**).

This new genus resembles *Medmassa* with similar males U-shaped sperm duct (Fig. 5A–C) and females spermathecae (Fig. 6A, B), but can be easily distinguished by the carapace bulge, highest before the fovea (Fig. 6D; carapace broad and flat in *Medmassa*), by the venter of the abdomen with large tracheal tubercle, covered with short, stout spines posteriorly (Fig. 6F; absent in *Medmassa*), by the embolus spine-shaped (Fig. 5A–C; embolus partial triangle in *Medmassa*), by the tibia with a spine-shaped ventral apophysis and without a retrolateral apophysis (Fig. 5A–C; tibia without ventral apophysis but with bifurcated retrolateral apophysis in *Medmassa*), by the epigyne with one copulatory opening (Fig. 6A; epigyne usually with two copulatory openings in *Medmassa*), and by the epigynal plate posteriorly with one membranous, nearly round hood (Fig. 6A; absent in most *Medmassa*). The new genus also resembles *Allomedmassa* with similar bulged carapace (Fig. 6D) and males spine-shaped tibial apophysis (Fig. 5A–C), but can be easily distinguished by the venter of the abdomen with a large tracheal tubercle, covered with short, stout spines posteriorly (Fig. 6F; absent in *Allomedmassa*), by the embolus slender and spine-shaped (Fig. 5A–C; embolus thick and most S-shaped in *Allomedmassa*), by the palpal tibia longer than wide and cylindrical (Fig. 5A–C; palpal tibia wider than long in *Allomedmassa*), by the epigyne with one small copulatory opening (Fig. 6A; epigyne with two large copulatory openings and clearly separated in *Allomedmassa*), by the epigynal plate posteriorly with one membranous, nearly round hood (Fig. 6A; absent in *Allomedmassa*), by the spermathecae large and elongate-elliptical (Fig. 6B; spermathecae small and reniform in *Allomedmassa*), by the copulatory ducts slender and tubular (Fig. 6B; copulatory ducts curved and thick in *Allomedmassa*), and by the vulva without an accessory gland (Fig. 6B; present in *Allomedmassa*).

##### Description.

Small-sized, non-ant-mimicking spiders (Fig. 6C–F). Carapace reddish brown to yellowish, obviously convex, highest before fovea, with dark marginal patterns; thoracic region almost round, cephalic region parallel-sided, widest at coxae II, gradually narrowing backwards; radial and cervical grooves indistinct; fovea longitudinal, dark brown. AER almost straight in frontal view, PER procurved in dorsal view; diameters of eyes almost same. MOA almost square. Clypeus height larger than diameter of AME. Chilum present, single, triangular, sclerotized. Chelicerae same color as carapace, with granular protrusions on surface, covered with short setae; with promarginal teeth and retromarginal teeth. Endites longer than wide, subapically with membranous area, apical margin with long, curved setae. Labium wider than long. Sternum shield-shaped, longer than wide. Legs brown to yellowish. Abdomen ovoid, grey; males with dorsal scutum, females without dorsal scutum; venter posteriorly with brown elliptical tracheal spiracle, covered with short, stout spines posteriorly.

Palpal (Fig. 5A–C) tibia short, longer than wide, covered with numerous bristles, and with slender spines dorsally; prolateral tibial tubercle triangular; ventral tibial apophysis, located in middle of tibia and spine-shaped. Cymbium long and narrow, with deep furrow ventrally, extending to tip. Tegulum elongate-elliptical, with U-shaped sperm duct. Subtegulum exposed prolaterally. Embolus slender, spine-shaped, and almost straight.

Epigynal region (Fig. 6A, B) heavily sclerotized. Epigynal plate round, as wide as long, posteriorly with one membranous, nearly round hood. Vulva with large, elongate-elliptical spermathecae close to each other, pair of fertilization ducts, and with two copulatory ducts converging into one copulatory opening.

##### Distribution.

China (Yunnan, Fig. 14).

##### Discussion.

This new genus *Fengzhen* can be easily distinguished from other genera in the family Corinnidae based on the following two most obvious morphological characteristics: male tibial apophysis located on the ventral surface and female two copulatory ducts converging into one copulatory opening. Morphologically, it is most similar to *Medmassa*, with the type species *Fengzhenmengla* sp. nov. being the most similar to *M.diplogale* Deeleman-Reinhold, 2001 from Borneo. The male palpal embolus and sperm duct of *M.diplogale* are similar to *F.mengla* sp. nov. but lack a prominent ventral tibial apophysis (Deeleman-Reinhold 2001: figs 544–546). At the same time, the hood on the epigynal plate posteriorly is a common feature of *M.diplogale* and *F.mengla* sp. nov., and *M.diplogale* with two obvious copulatory openings (Deeleman-Reinhold 2001: figs 553, 554). None of the other females in *Medmassa* have hoods. Another important feature that distinguishes *Fengzhen* from *Medmassa* is that the former has a bulging carapace, while the latter has a flat carapace. Based on the above morphological characteristics, we consider the species from Xishuangbanna, China as a new genus *Fengzhen* gen. nov. Due to the specimen being stored at room temperature for more than ten years, DNA extraction is no longer possible, so cladistics analysis is missing in this work. Therefore, the monophyly of *Fengzhen* needs further discussion in future work that include molecular analysis.

#### ﻿ Fengzhen mengla

Taxon classificationAnimaliaAraneaeCorinnidae

Lu & Li sp. nov.

268D734B-673B-5B29-8A8A-0B8AEC94F599

https://zoobank.org/E917C848-EAD3-41C3-AA46-EB274D81D824

Figs 5 , 6

##### Type material.

***Holotype***: 1♂ (IZCAS-Ar 44420), **China**, Yunnan, Xishuangbanna, Mengla County, Menglun Town, Menglun Botanical Garden, Lvshilin, 21°54.609′N, 101°16.871′E, 663 m, hand catch in leaf litter, 14 November 2009, G. Tang and Z.Y. Yao leg. ***Paratypes***: 1♂ (IZCAS-Ar 44421) and 1♀ (IZCAS-Ar 44422), same data as holotype.

##### Etymology.

The specific name refers to the type locality and is a noun in apposition.

##### Diagnosis.

See the generic diagnosis above.

##### Description.

**Male** (**holotype**; Fig. 6C, D). Total body length 4.33: carapace 2.21 long, 1.66 wide; abdomen 2.12 long, 1.43 wide. Carapace reddish brown, obviously convex, highest before fovea, with dark marginal patterns; thoracic region almost round, cephalic region parallel-sided; radial and cervical grooves indistinct; fovea longitudinal, dark brown. Diameters of eyes: AME 0.14, ALE 0.13, PME 0.13, PLE 0.12. Eye interdistances: AME–AME 0.10, AME–ALE 0.05, PME–PME 0.13, PME–PLE 0.11, AME–PME 0.14, ALE–PLE 0.04. CRW/carapace width = 0.67. MOA 0.38 long, front width 0.32, back width 0.37. Clypeus height almost 1.5 ×diameter of AME. Chilum present, single, triangular, sclerotized and reddish brown. Chelicerae same color as carapace, with granular protrusions on surface, covered with short setae; with three promarginal teeth, six retromarginal teeth. Endites brown, longer than wide, subapically with membranous area, apical margin with long, curved setae. Labium brown, 0.28 long, 0.40 wide. Sternum brownish, shield-shaped, longer than wide. Sternum 0.98 long, 1.00 wide. Legs brown, but brownish on coxae. Measurements of legs: I – (1.67, 0.67, 1.34, 1.07, –), II 5.41 (1.52, 0.62, 1.18, 1.11, 0.98), III 5.33 (1.45, 0.62, 1.11, 1.26, 0.89), IV 6.19 (1.73, 0.64, 1.34, 1.69, 0.79). Leg spination: tibiae I with six pairs of ventral spines, II with four pairs of ventral spines, III and IV with three pairs of ventral spines; metatarsi I–III with two pairs of ventral spines, IV with three pairs of ventral spines. Abdomen ovoid, grey, with short purple stripes and reddish brown scutum covering 3/4 of dorsum surface, covered with black spots; venter yellowish, posteriorly with brown elliptical tracheal spiracle, covered with short, stout spines posteriorly. Spinnerets yellowish.

***Palp*** (Fig. 5A–C). Tibia with triangular prolateral tibial tubercle and spine-shaped ventral tibial apophysis, located in middle of tibia; retrolateral tibial apophysis absent. Cymbium long and narrow, retrolaterally with small outgrowth, and with deep furrow ventrally, extending to tip. Tegulum elongate-elliptical, 3/4 length of cymbium, with U-shaped sperm duct. Subtegulum exposed prolaterally. Embolus slender, spine-shaped, and almost straight.

**Female** (**paratype**; Fig. 6E, F). Total body length 4.68: carapace 1.95 long, 1.56 wide; abdomen 2.73 long, 1.87 wide. Color and somatic morphology as in male, except as noted. Carapace yellowish. Diameters of eyes: AME 0.12, ALE 0.10, PME 0.11, PLE 0.12. Eye interdistances: AME–AME 0.07, AME–ALE 0.04, PME–PME 0.10, PME–PLE 0.10, AME–PME 0.13, ALE–PLE 0.06. CRW/carapace width = 0.59. MOA 0.34 long, front width 0.27, back width 0.30. Chilum brown. Endites, labium and sternum yellowish. Labium 0.26 long, 0.37 wide. Sternum 0.99 long, 0.96 wide. Measurements of legs: I 5.52 (1.62, 0.63, 1.27, 1.04, 0.96), II 5.17 (1.46, 0.56, 1.15, 1.07, 0.93), III 5.30 (1.44, 0.56, 1.11, 1.30, 0.89), IV 6.39 (1.73, 0.60, 1.43, 1.83, 0.80). Abdomen grey, with lavender spots and without dorsal scutum.

***Epigyne*** (Fig. 6A, B). Epigynal plate round, as wide as long, posteriorly with one membranous, nearly round hood. Vulva with large, elongate-elliptical spermathecae close to each other, and pair of fertilization ducts pointing antero-laterally. Copulatory opening round, situated at anterior part of epigynal plate. Copulatory ducts long and tubular, situated in middle of vulva.

##### Variation.

Paratype male: total body length 4.51.

##### Distribution.

China (Yunnan, type locality; Fig. 14).

#### ﻿Genus Medmassa

Taxon classificationAnimaliaAraneaeCorinnidae

Simon, 1887

5C2203F1-6F8C-5B2E-929C-1C2078EE45BF

##### Type species.

*Megaerafrenata* Simon, 1877 from Philippines.

##### Composition.

A genus encompassing ten species, covering Africa, Asia, and Oceania. Of these, six species are from Southeast Asia: *M.celebensis* (Deeleman-Reinhold, 1995) (♀) from Indonesia, *M.frenata* (juvenile) from Philippines, *M.insignis* (Thorell, 1890) (♂♀) from Indonesia, *M.kltina* (Barrion & Litsinger, 1995) (♀) from Philippines, *M.tigris* (Deeleman-Reinhold, 1995) (♂♀) from Indonesia, and *M.torta* Jin, Zhang & Zhang, 2019 (♂) from China.

#### ﻿ Medmassa lingshui

Taxon classificationAnimaliaAraneaeCorinnidae

Lu & Li sp. nov.

114BAB6C-211B-53AF-BA40-E6014E68E573

https://zoobank.org/6C6F70A8-7442-4B48-9881-DDEDA3548DF5

Figs 7 , 8

##### Type material.

***Holotype***: 1♂ (IZCAS-Ar 44423), **China**, Hainan, Lingshui, Diaoluoshan Mountain, 18°40.440′N, 109°52.600′E, 505 m, hand catch in leaf litter, 10 August 2010, G. Zheng leg.

##### Etymology.

The specific name refers to the type locality and is a noun in apposition.

##### Diagnosis.

**Figure 7.**
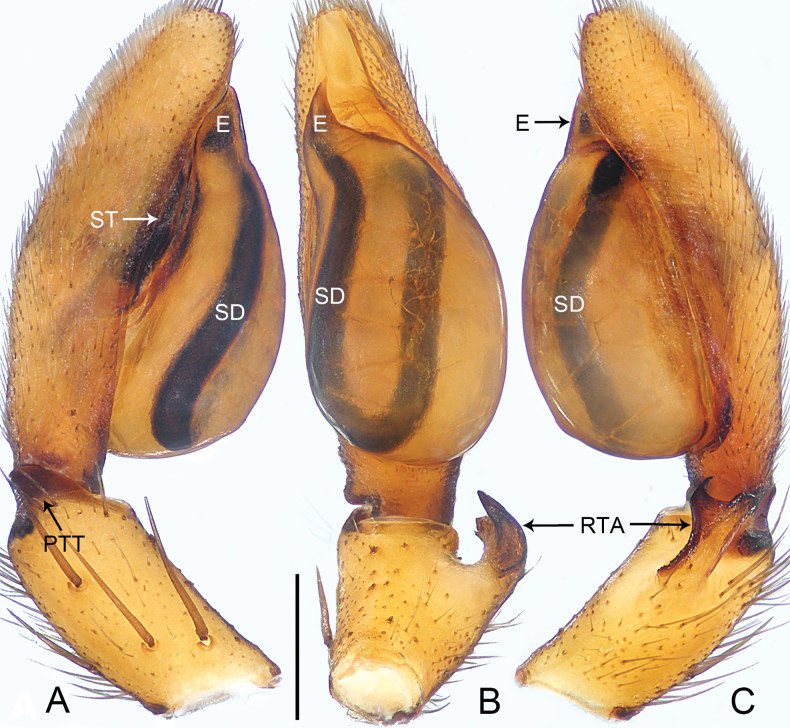
*Medmassalingshui* sp. nov., holotype male **A–C** palp **A** prolateral view **B** ventral view **C** retrolateral view. Abbreviations: E = embolus, PTT = prolateral tibial tubercle, RTA = retrolateral tibial apophysis, SD = sperm duct, ST = subtegulum. Scale bar: 0.50 mm.

**Figure 8.**
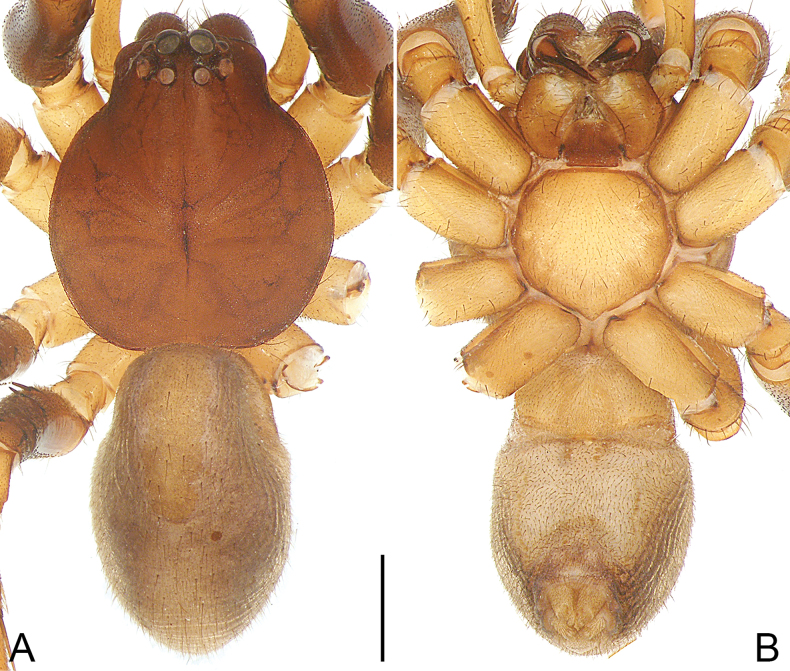
*Medmassalingshui* sp. nov., holotype male **A, B** habitus **A** dorsal view **B** ventral view. Scale bar: 1.00 mm.

The new species resembles *M.torta* Jin, Zhang & Zhang, 2019 (cf. Figs 7, 8 and Jin et al. 2019: 469, figs 8A–H, 9A–C) as the males have a similar sclerotized, triangular prolateral tibial tubercle (Fig. 7A) and small prolatero-proximal cymbial outgrowth (Fig. 7B). Males can be distinguished by the triangular embolus, that is slightly curved, and integrated with the tegulum (Fig. 7B; vs. spine-shaped embolus, almost straight), by the retrolateral tibial apophysis entirely sclerotized apically (Fig. 7B, C; vs. retrolateral tibial apophysis twisted and weakly sclerotized), and by the ventral branch of the retrolateral tibial apophysis pointed and curved, and the dorsal branch of the retrolateral tibial apophysis blunt, pointing dorsally (Fig. 7B, C; vs. ventral and dorsal branches of retrolateral tibial apophysis almost straight, pointing ventrally). Female unknown.

##### Description.

**Male** (**holotype**; Fig. 8A, B). Total body length 6.30: carapace 2.97 long, 2.69 wide; abdomen 3.33 long, 1.91 wide. Carapace dark brown, smooth, almost round, and flat, strong arched caput, sloping gradually back from fovea; posterior margin truncated; fovea longitudinal, black. Diameters of eyes: AME 0.25, ALE 0.14, PME 0.16, PLE 0.17. Eye interdistances: AME–AME 0.10, AME–ALE 0.04, PME–PME 0.20, PME–PLE 0.12, AME–PME 0.15, ALE–PLE 0.11. CRW/carapace width = 0.52. MOA 0.54 long, front width 0.56, back width 0.51. Clypeus height 2 × AME diameter. Chilum present, single, and triangular. Chelicerae same color as carapace, without concave depression at distal end dorsally; with three promarginal teeth, four retromarginal teeth. Endites brown, longer than wide, subapically with membranous area, apical margin with long, curved setae. Labium dark brown, as wide as long. Labium 0.49 long, 0.55 wide. Sternum yellowish, with brown margin, shield-shaped, as wide as long. Sternum 1.49 long, 1.55 wide. Legs dark brown and strong, but yellowish on coxae and trochanters. Measurements of legs: I 10.32 (2.92, 1.16, 2.79, 2.21, 1.24), II 9.66 (2.73, 1.11, 2.35, 2.19, 1.28), III 10.47 (2.61, 1.08, 2.24, 2.87, 1.67), IV 12.12 (2.98, 1.14, 2.65, 3.44, 1.91). Leg spination: tibiae I with nine pairs of ventral spines, II with six pairs of ventral spines, III with two pairs of ventral spines, IV with three pairs of ventral spines; metatarsi I–IV with three pairs of ventral spines. Abdomen ovoid, dark grey, with yellowish dorsal scutum centrally; venter yellowish, posteriorly grey; laterally dark grey, with pale stripes. Spinnerets yellowish.

***Palp*** (Fig. 7A–C). Tibia with sclerotized, triangular prolateral tibial tubercle; retrolateral tibial apophysis distally bifurcated, ventral branch curved and pointed, dorsal branch sclerotized and blunt. Cymbium elongate-oval, dorsally with chemosensory patch, prolaterally with small outgrowth, and with deep furrow ventrally, extending to tip. Tegulum oval, 3/4 length of cymbium, with U-shaped sperm duct. Subtegulum exposed prolaterally. Embolus triangular and short, integrated with tegulum.

##### Distribution.

China (Hainan, type locality; Fig. 14).

#### ﻿Genus Peng

Taxon classificationAnimaliaAraneaeCorinnidae

Lu & Li gen. nov.

3E3EADDC-CADA-5531-8BC1-3F13C112CB42

https://zoobank.org/FF31429F-E8E2-452E-A515-BB5623DE00A9

##### Type species.

*Myrmeciscabirmanica* Thorell, 1897.

##### Composition.

This new genus includes three species: *P.birmanicus* (Thorell, 1897), comb. nov. (♂♀) from Myanmar and China, *P.borneensis* (Yamasaki, 2017), comb. nov. (♂♀) from Malaysia (Borneo) and *P.taprobanicus* (Simon, 1897), comb. nov. (juvenile) from Sri Lanka.

##### Etymology.

The generic name is dedicated to Chinese arachnologist Xianjin Peng, born in 1963 in Cili, Hunan Province, China. Gender is masculine.

##### Diagnosis.

**Figure 9.**
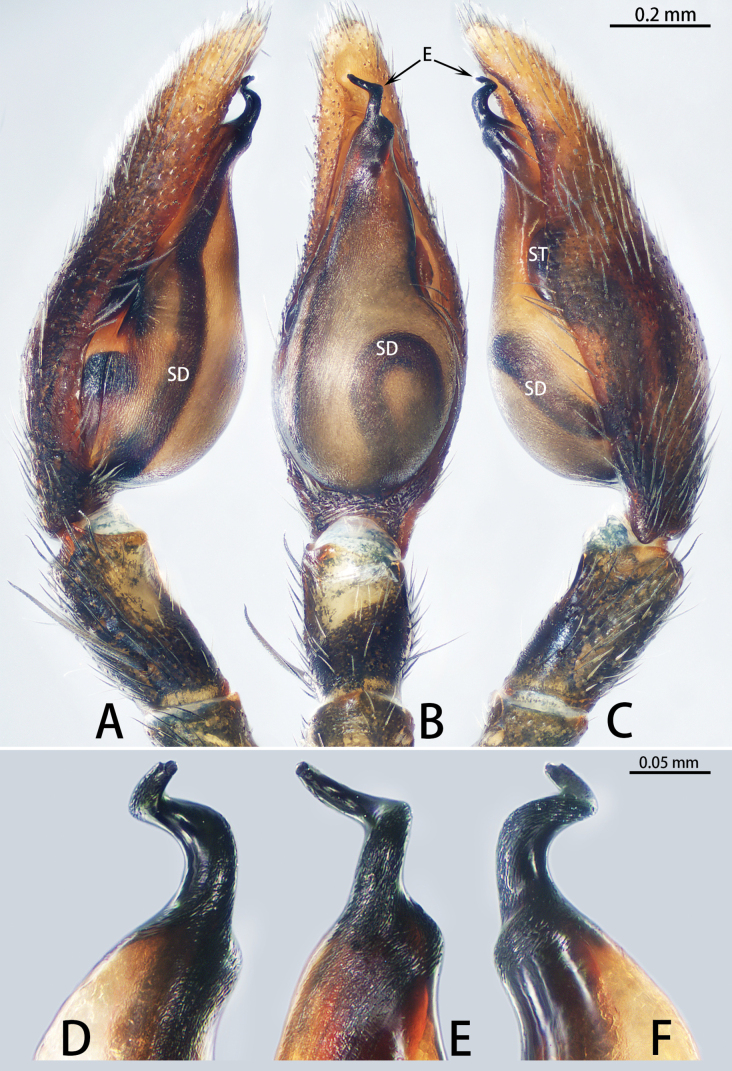
*Pengbirmanicus* comb. nov., male **A–C** palp, **D–F** embolus **A** prolateral view **B** ventral view **C** retrolateral view **D** prolateral view **E** ventral view **F** retrolateral view. Abbreviations: E = embolus, SD = sperm duct, ST = subtegulum. Scale bars: 0.20 mm (**A–C**); 0.05 mm (**D–F**).

**Figure 10.**
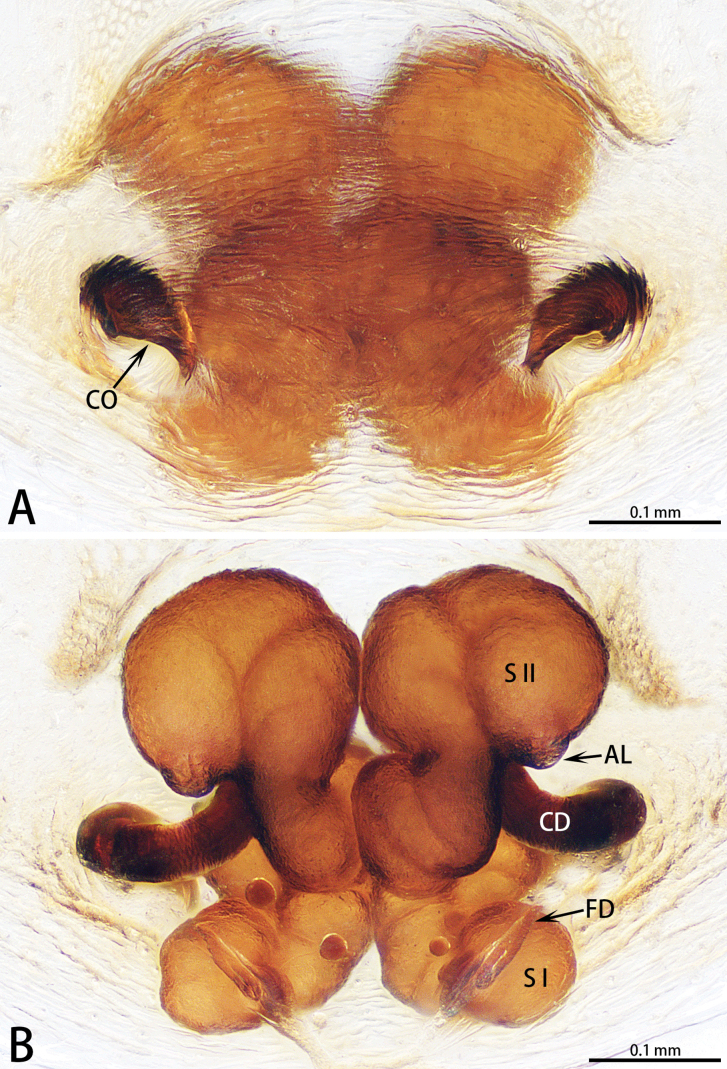
*Pengbirmanicus* comb. nov., female (**A, B**) **A** epigyne, ventral view **B** vulva, dorsal view. Abbreviations: AL = accessory lobe, CD = copulatory duct, CO = copulatory opening, FD = fertilization duct, S I = spermathecae I, S II = spermathecae II. Scale bars: 0.10 mm.

**Figure 11.**
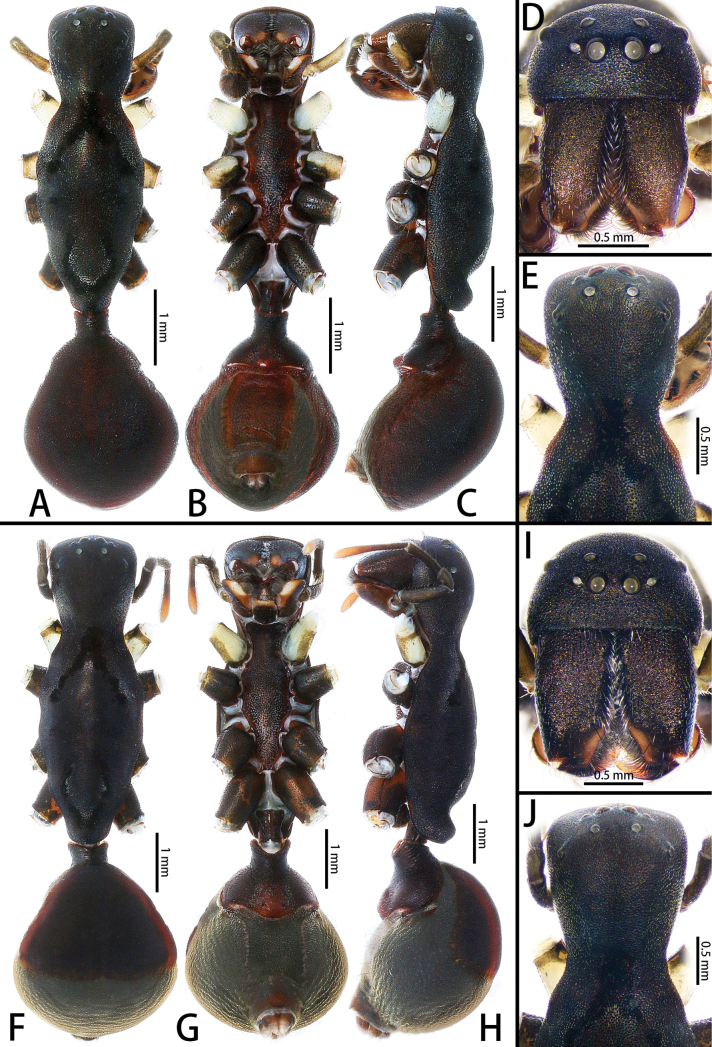
*Pengbirmanicus* comb. nov., male (**A–E**) and female (**F–J**) **A** habitus, dorsal view **B** habitus, ventral view **C** habitus, lateral view **D** cephalothorax, frontal view **E** cephalic region, dorsal view **F** habitus, dorsal view **G** habitus, ventral view **H** habitus, lateral view **I** cephalothorax, frontal view **J** cephalic region, dorsal view. Scale bars: 1.00 mm (**A–C, F–H**); 0.50 mm (**D, E, I, J**).

This new genus can be easily distinguished from *Sphecotypus* by the carapace lateral margins weakly undulated (Fig. 11A, F; carapace lateral margins strongly undulated in *Sphecotypus*, Leister and Miller 2014: fig. 1A), by the sternum without the intercoxal sclerite between two coxae IV (Fig. 11B, G; present in *Sphecotypus*, Leister and Miller 2014: fig. 1B), by the abdomen ovoid, without median constriction (Fig. 11A–C, F–H; abdomen divided into two lobes by strong median constriction, anterior lobe spherical, posterior lobe elliptical in *Sphecotypus*, Leister and Miller 2014: fig. 1A–C), by the tibia with triangular prolateral tibial tubercle, without retrolateral apophysis and setal projection (Fig. 9A–C; triangular prolateral tibial tubercle absent, retrolateral apophysis and setal projection present in *Sphecotypus*, see Leister and Miller 2014: fig. 1D–F), by the sperm duct curved twice at ventral surface of posterior tegulum (Fig. 9A–C; sperm duct curved 4 × at ventral surface of posterior tegulum in *Sphecotypus*, see Leister and Miller 2014: fig. 1D–F), by the embolus short, twisted, apically hook-shaped (Fig. 9A–F; embolus conical, with fine screw-like wrinkles in *Sphecotypus*, see Leister and Miller 2014: fig. 1D–G), and by the spermathecae symmetrical (Fig. 10B; spermathecae asymmetrical in *Sphecotypus*, see Leister and Miller 2014: fig. 1I).

##### Description.

Small-sized, ant-mimicking spiders (Fig. 11A–J). Carapace black, covered with granular protuberances, with two distinct regions, cephalic region ladder-shaped, distinguished from thoracic region by deep constriction, thoracic region long, almost 2 × of cephalic region, fusiform, lateral margins weakly undulated, terminating with small raised dome; thoracic groove absent. AER slightly procurved in frontal view, PER strongly recurved in dorsal view; AME largest, diameter of ALE subequal to PLE. MOA almost square. Clypeus height larger than diameter of AME. Chelicerae same color as carapace, covered with long dark setae along anterior surface, and with intensive promarginal setae. Endites brown to black, longer than wide, subapically with membranous area, apical margin with long setae. Labium black, longer than wide. Sternum reddish brown to black, elongate, granulose, covered with white feathery setae, anteriorly extending beyond coxae I, tapering posteriorly, extending between coxae IV, contiguous with precoxal and intercoxal sclerites. Sternum much longer than wide. Legs black, but white on most coxae I. Abdomen ovoid, reddish brown to black, covered with granular protuberances, males with scutum almost covering the whole dorsum surface, females with scutum almost covering 1/2 to 2/3 of dorsum surface.

Palpal (Fig. 9A–F) tibia short, longer than wide, covered with numerous bristles, and with slender spines prolaterally; prolateral tibial tubercle triangular; without retrolateral apophysis and setal projection. Cymbium tip conical, with deep furrow ventrally. Tegulum pyriform, with sperm duct curved twice at ventral surface of posterior tegulum. Subtegulum exposed retrolaterally. Embolus short, sclerotized, strongly curved apically.

Epigynal region (Fig. 10A, B) heavily sclerotized. Epigynal plate with two elliptical, downward copulatory openings, situated at posterior part of epigynal plate. Vulva with symmetrical spermathecae, divided into two chambers, shape of spermathecae varies. Copulatory ducts tubular, connect the junction of two chambers.

##### Distribution.

China (Yunnan), Myanmar, Malaysia (Borneo) and Sri Lanka.

##### Note.

According to the clear figures in Yamasaki and Rollard (2022), the somatic morphology of *Sphecotypustaprobanicus* Simon, 1897 conforms to *Peng* gen. nov. Therefore, *S.taprobanicus* is transferred to *Peng*, as *Pengtaprobanicus* (Simon, 1897) comb. nov.

##### Discussion.

The genus *Sphecotypus* was established based on a species collected from Nicaragua to Brazil and Bolivia by O. Pickard-Cambridge in 1895. Subsequently, three species from Asia were added to this genus. From a morphological perspective, there are significant differences in habitus and genitals between American and Asian species, such as the median constriction of abdomen, intercoxal sclerite between two coxae IV on the sternum and male palp (refer to the above genus diagnosis for details). Secondly, combined with geographical distribution, we transferred three species from Asia and established a new genus *Peng* gen. nov. Due to the fact that the specimens collected at that time were not stored at low temperature in 95% alcohol, DNA could no longer be extracted. The phylogenetic relationship between *Peng* and other related genera needs further experimental discussion.

#### ﻿ Peng birmanicus

Taxon classificationAnimaliaAraneaeCorinnidae

(Thorell, 1897) comb. nov.

33663607-501E-5348-B797-C134702524A8

Figs 9 , 10 , 11

Myrmecisca birmanica Thorell, 1897: 240; SphecotypusbirmanicusSimon 1897: 171; Yamasaki et al. 2017: 22, figs 1–5, 7–8; Yamasaki and Rollard 2022: 49, fig. 2B, E.

##### Material examined.

1♂ (IZCAS-Ar 44424) and 1♀ (IZCAS-Ar 44425), **China**, Yunnan, Xishuangbanna, Mengla County, Menglun Town, Xishuangbanna Botanical Garden, 21°55′16.6′′N, 101°16′35.4′′E, 564 m, hand catch in leaf litter, 7 April 2015, Z.G. Chen leg.

##### Diagnosis.

The new species resembles *P.borneensis* (Yamasaki, 2017) (cf. Figs 9–11 and Yamasaki et al. 2017: 26, figs 9–28) as the males have a similar sperm duct (Fig. 9A–C). Males can be distinguished by the embolus slightly curved apically in ventral view (Fig. 9B, E; vs. embolus strongly curved apically in ventral view); females by the copulatory ducts long and curved (Fig. 10B; vs. copulatory ducts shorter and almost straight), by the spermathecae II comma-shaped (Fig. 10B; vs. spermathecae II large and sac-like), and by the spermathecae II with accessory lobes (Fig. 10B; vs. spermathecae II without accessory lobes).

##### Description.

**Male** (Fig. 11A–E). Total body length 6.79: carapace 3.94 long, 1.48 wide; abdomen 2.85 long, 1.96 wide. Carapace black, covered with granular protuberances, with two distinct regions, cephalic region ladder-shaped, distinguished from thoracic region by deep constriction, thoracic region long, fusiform, lateral margins weakly undulated, terminating with small raised dome; thoracic groove absent. Diameters of eyes: AME 0.13, ALE 0.08, PME 0.10, PLE 0.09. Eye interdistances: AME–AME 0.13, AME–ALE 0.05, PME–PME 0.23, PME–PLE 0.30, AME–PME 0.15, ALE–PLE 0.29. CRW/carapace width = 0.74. MOA 0.36 long, front width 0.41, back width 0.43. Clypeus height almost 1.5 × diameter of AME. Chelicerae same color as carapace, covered with long dark setae along anterior surface; with two promarginal teeth, two retromarginal teeth. Endites brown to black, longer than wide, subapically with membranous area, apical margin with long setae. Labium black, 0.45 long, 0.35 wide. Sternum reddish brown to black, elongate, granulose, covered with white feathery setae, anteriorly extending beyond coxae I, tapering posteriorly, extending between coxae IV, contiguous with precoxal and intercoxal sclerites. Sternum 1.94 long, 0.81 wide. Legs black, but white on coxae I and II, with black bands. Measurements of legs: I 8.52 (2.35, 0.51, 2.38, 1.97, 1.31), II 7.60 (2.24, 0.57, 2.07, 1.65, 1.07), III 6.60 (2.02, 0.63, 1.68, 1.60, 0.67), IV 10.22 (3.29, 0.68, 2.58, 2.61, 1.06). Leg spination: metatarsi I with 1 ventral spine, II–III with two pairs of ventral spines, IV with 1 pair of ventral spines. Abdomen ovoid, reddish brown to black, covered with granular protuberances; venter anteriorly with black epigastric sclerite, reddish brown rectangular ventral sclerite, posteriorly with brown spiracle. Spinnerets brown in anterior part, white in posterior part.

***Palp*** (Fig. 9A–C). Tibia with triangular prolateral tibial tubercle. Cymbium tip conical, with deep furrow ventrally. Tegulum pyriform, 4/5 length of cymbium, with distinct, sinuous sperm duct. Subtegulum exposed retrolaterally. Embolus short, sclerotized, twisted, apically hook-shaped.

**Female** (Figs 10A, B, 11F–J). See Yamasaki et al. (2017: figs 1–5, 7–8) and Yamasaki and Rollard (2022: fig. 2B, E) for complete description.

##### Distribution.

Myanmar (type locality); China (Yunnan; Fig. 14).

#### ﻿Genus Spinirta

Taxon classificationAnimaliaAraneaeCorinnidae

Jin & Zhang, 2020

4BECA08D-5054-5A88-BB85-E28313818D84

##### Type species.

*Spinirtajinyunshanensis* Jin & Zhang, 2020 from China.

##### Composition.

The genus is endemic to China, and 17 species are currently included: *S.aurita* Jin & Zhang, 2020 (♂), *S.aviforma* Jin & Zhang, 2020 (♂), *S.caudata* Zhang, Jin & Zhang, 2023 (♂), *S.forcipata* Jin & Zhang, 2020 (♂♀), *S.jinyunshanensis* Jin & Zhang, 2020 (♂♀), *S.lanceolata* Zhang, Jin & Zhang, 2023 (♂), *S.leigongshanensis* Jin & Zhang, 2020 (♂♀), *S.qiaoliaoensis* (Lu & Chen, 2019) (♂♀), *S.qishuoi* Lin & Li, 2023 (♀), *S.qizimeiensis* Jin & Zhang, 2020 (♀), *S.quadrata* Jin & Zhang, 2020 (♂), *S.rugosa* Jin & Zhang, 2020 (♂♀), *S.sanxiandian* Liu, 2022 (♂♀), *S.shenwushanensis* Zhang, Jin & Zhang, 2023 (♂♀), *S.sishuishan* Liu, 2022 (♂), *S.sparsula* Jin & Zhang, 2020 (♂♀), *S.wuyishanensis* Zhou, 2022 (♂).

#### ﻿ Spinirta shaoguan

Taxon classificationAnimaliaAraneaeCorinnidae

Lu & Li sp. nov.

9341B18A-713D-5BA1-B475-36F5E9BA7DF3

https://zoobank.org/DAE661B6-5742-42BF-8EC8-3DB835BF5EF5

Figs 12 , 13

##### Type material.

***Holotype***: 1♂ (IZCAS-Ar 44426), **China**, Guangdong, Shaoguan, Nanling Nature Reserve, 24.9287°N, 113.0102°E, hand catch in leaf litter, 16–21 July 2008, G. Tang leg.

##### Etymology.

The specific name refers to the type locality and is a noun in apposition.

##### Diagnosis.

**Figure 12.**
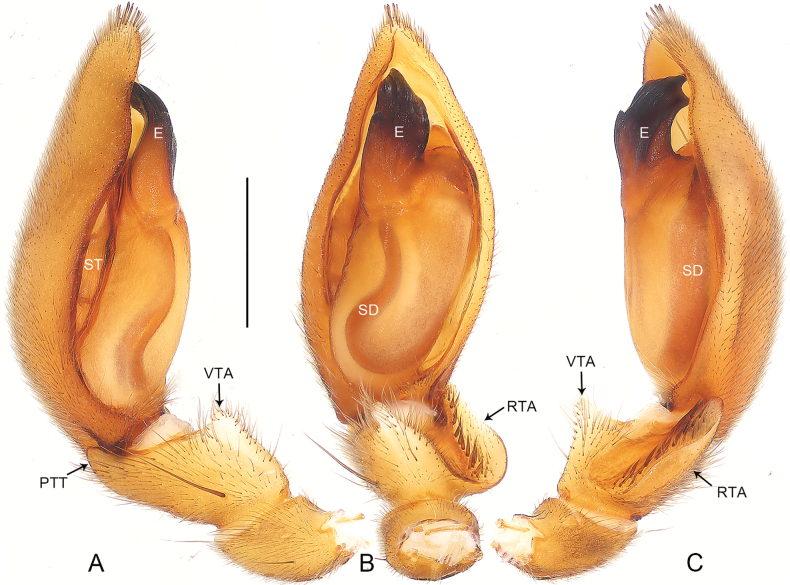
*Spinirtashaoguan* sp. nov., holotype male **A–C** palp **A** prolateral view **B** ventral view **C** retrolateral view. Abbreviations: E = embolus, PTT = prolateral tibial tubercle, RTA = retrolateral tibial apophysis, SD = sperm duct, ST = subtegulum, VTA = ventral tibial apophysis. Scale bar: 1.00 mm.

**Figure 13.**
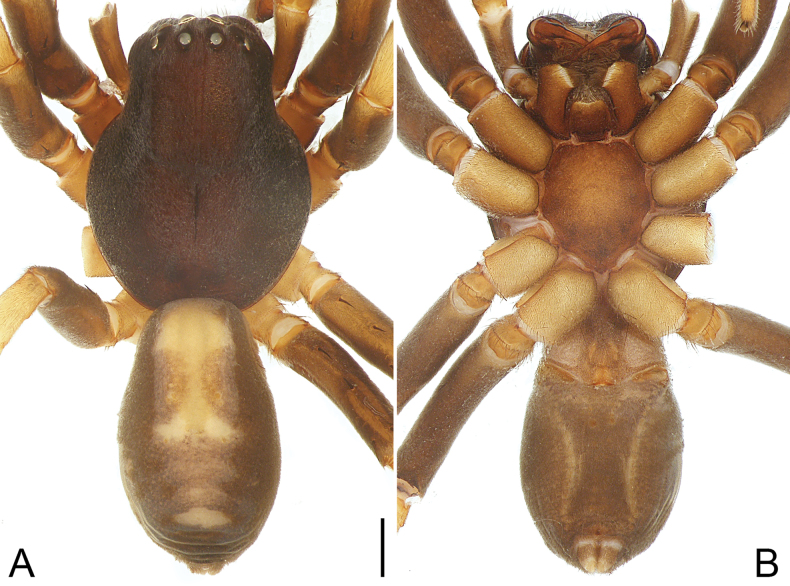
*Spinirtashaoguan* sp. nov., holotype male **A, B** habitus **A** dorsal view **B** ventral view. Scale bar: 1.00 mm.

The new species resembles *S.aurita* Jin & Zhang, 2020 (cf. Figs 12, 13 and Jin and Zhang 2020: 317, figs 3D, 14A–I, 15A–D) as the males have similar triangular prolateral tibial tubercle (Fig. 12A) and a retrolateral tibial apophysis with the outer edge ear-shaped (Fig. 12B, C). Males can be distinguished by the tibia with a large, triangular ventral apophysis (Fig. 12A–C; vs. tibia with inconspicuous ventral protrusion), by the ventral surface of retrolateral tibial apophysis with long coniform spines (Fig. 12B, C; vs. ventral surface of retrolateral tibial apophysis with short coniform spines), and by the embolus digitiform, curved, apically sclerotized, with file-like grooves on the surface, and with two coniform apophyses distally and a small sharp apophysis centrally (Fig. 12A–C; vs. embolus long, with long and sharp embolar apophysis, file-like grooves almost invisible on embolar apophysis surface). Female unknown.

##### Description.

**Male** (**holotype**, Fig. 13A, B). Total body length 9.64: carapace 5.05 long, 3.86 wide; abdomen 4.59 long, 2.82 wide. Carapace dark brown to black, convex, with rough surface; highest before fovea; thoracic region ovoid, cephalic region with parallel sides; widest at coxae II, gradually narrowing backwards, slightly concave at posterior margin before pedicel; radial and cervical grooves indistinct; fovea longitudinal, short. Diameters of eyes: AME 0.19, ALE 0.22, PME 0.20, PLE 0.23. Eye interdistances: AME–AME 0.30, AME–ALE 0.21, PME–PME 0.33, PME–PLE 0.38, AME–PME 0.34, ALE–PLE 0.17. CRW/carapace width = 0.61. MOA 0.70 long, front width 0.62, back width 0.73. Clypeus height narrower than diameter of AME. Chilum present, single, triangular, sclerotized, and brown. Chelicerae same color as carapace; with three promarginal teeth, five retromarginal teeth. Endites brown, longer than wide, subapically with membranous area, apical margin with long, curved setae. Labium dark brown, longer than wide. Labium 0.82 long, 0.73 wide. Sternum dark brown marginally and light brown centrally, shield-shaped, longer than wide, precoxal triangles present. Sternum 2.26 long, 1.85 wide. Legs dark brown to yellowish, coxae I brown, coxae II–IV yellowish. Measurements of legs: I 14.76 (4.11, 1.83, 3.58, 3.39, 1.85), II 14.09 (3.98, 1.72, 3.32, 3.29, 1.78), III 11.92 (3.28, 1.56, 2.69, 2.82, 1.57), IV 15.29 (3.94, 1.69, 3.56, 4.45, 1.65). Leg spination: tibiae I–II with four pairs of ventral spines, III–IV with two pairs of ventral spines; metatarsi I–IV with two pairs of ventral spines. Abdomen ovoid, dark brown, with longitudinal strip and yellowish patch anteriorly and medially, and white patch posteriorly; venter dark brown, with two yellowish arched patches. Spinnerets yellowish, with brown marks.

***Palp*** (Fig. 12A–C). Tibia with triangular prolateral tibial tubercle and large, triangular ventral tibial apophysis; retrolateral tibial apophysis outer edge ear-shaped, ventral surface with dense thick long coniform spines. Cymbium tip conical, apically with stout setae, and with deep furrow ventrally. Tegulum elongate oval, 2/3 length of cymbium, with U-shaped sperm duct, sinuous at distal part. Subtegulum exposed prolaterally. Embolus digitiform, curved, apically sclerotized, with file-like grooves on surface, and with two coniform apophyses distally and small sharp apophysis centrally.

##### Distribution.

**Figure 14.**
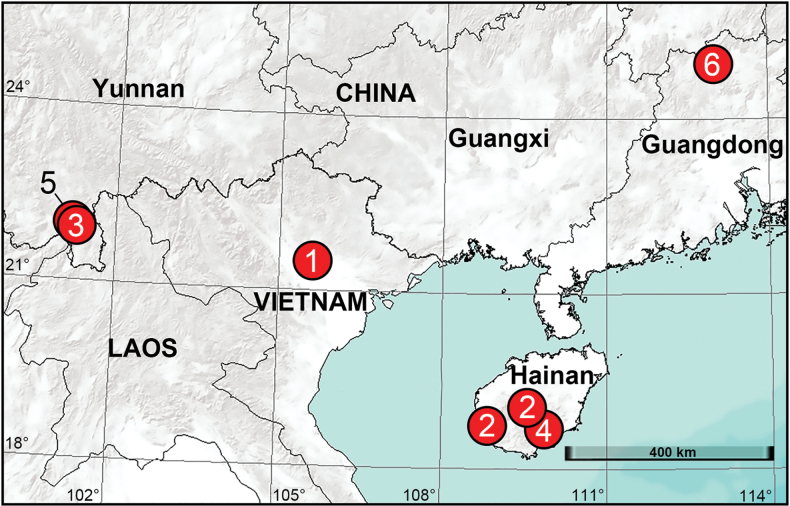
New distribution records of corinnid species from China and Vietnam **1***Allomedmassatamdao* sp. nov. **2***Echinaxbaisha* sp. nov. **3***Fengzhenmengla* sp. nov. **4***Medmassalingshui* sp. nov. **5***Pengbirmanicus* comb. nov. **6***Spinirtashaoguan* sp. nov.

China (Guangdong, type locality; Fig. 14).

## Supplementary Material

XML Treatment for Allomedmassa

XML Treatment for Allomedmassa tamdao

XML Treatment for Echinax

XML Treatment for Echinax baisha

XML Treatment for Fengzhen

XML Treatment for Fengzhen mengla

XML Treatment for Medmassa

XML Treatment for Medmassa lingshui

XML Treatment for Peng

XML Treatment for Peng birmanicus

XML Treatment for Spinirta

XML Treatment for Spinirta shaoguan

## ﻿Additional information

### Author ORCIDs

Ying Lu https://orcid.org/0000-0002-0316-3564

Chang Chu https://orcid.org/0000-0003-3520-5463

Dinh-Sac Pham https://orcid.org/0000-0001-8594-5270

Shuqiang Li https://orcid.org/0000-0002-3290-5416

Zhiyuan Yao https://orcid.org/0000-0002-1631-0949

### Data availability

All of the data that support the findings of this study are available in the main text or Supplementary Information.
